# Body Shape Index and Cardiovascular Risk in Individuals With Obesity

**DOI:** 10.7759/cureus.21259

**Published:** 2022-01-14

**Authors:** Nazlı Hacıağaoğlu, Can Öner, Hüseyin Çetin, Engin Ersin Şimşek

**Affiliations:** 1 Family Medicine, S.B.Ü. Kartal Dr. Lütfi Kırdar City Hospital, İstanbul, TUR

**Keywords:** score, bmi, absi, cardiovascular risk, obesity

## Abstract

Introduction: Cardio-metabolic risks are tested to show various anthropometric measurements. This study aimed to evaluate a body shape index (ABSI) of individuals with obesity to determine the role of these measurements in cardiovascular risk prediction.

Methods: This cross-sectional study included 368 patients who were evaluated by the researcher in the polyclinic. Sociodemographic information was obtained, and anthropometric measurements were made. Body mass index (BMI), waist circumference, and ABSI were evaluated in all patients. The patient’s risk of developing cardiovascular disease was calculated from the pooled cohort equations risk calculator (PRCAE), Framingham risk score, and systematic coronary risk evaluation (SCORE) risk calculation systems.

Results: Of the 368 patients in the study, 302 (82.1%) were females, and 66 (17.9%) were males. The average age of participants was 46.2 ± 12.0 years. The median BMI of participants was 37.5 (34.0-42.4) kg/m^2^. The median ABSI of participants was 0.0816 (0.0775-0.0849). A positive correlation was found between ABSI and Framingham risk score and PRCAE risk score (r = 0.297, *p* = 0.000 and r = 0.305, *p* = 0.000, respectively). A significant relationship was found between ABSI and Framingham, PRCAE, and SCORE risk groups (*p* = 0.000, *p* = 0.000, and *p* = 0.000, respectively).

Conclusions: Our study results revealed a significant association of ABSI with Framingham, PRCAE, and SCORE risk calculation systems, which helps predict cardiovascular risk.

## Introduction

Obesity is one of the most serious health problems [1] and is a risk factor for many diseases worldwide [2]. The World Health Organization states that >650 million adults have obesity, and its prevalence is increasing annually [3]. Obesity, which can begin in childhood, is associated with all risk factors, such as hypertension, hyperlipidemia, insulin resistance, and diabetes mellitus, which can lead to cardiometabolic diseases in adulthood. Body mass index (BMI) with the calculation of body weight (kg)/height square meter (m)^2^ is often used in the definition of obesity. BMI indicates patients at high cardiometabolic risk [1]. However, BMI is insufficiently successful in reflecting fat distribution and distinguishing between muscle and fat mass [4]. Studies show that waist circumference measurement is more valuable in determining abdominal obesity [4,5].

Various anthropometric measurements tried to more clearly show the cardio-metabolic risk. These include waist circumference, waist-to-height ratio, and waist-hip ratio, as well as a body shape index (ABSI), which has been researched in recent years [1]. A study revealed that the measurements calculated with ABSI were more related to cardiovascular risk than BMI [6]. Unlike BMI, ABSI is calculated according to age and gender [5]. Anthropometric parameters are non-invasive and cost-effective, and reliable parameters for cardiovascular diseases and associated risk factors [7]. ABSI has been associated with an increased risk of mortality and premature death in adults [1,7].

One of the most important steps in preventing cardiovascular diseases and premature deaths is risk identification and correction of preventable risk factors. Therefore, screening patients with obesity, who are an at-risk group for cardiovascular diseases, is important. This study aimed to determine the role of ABSI measurements in predicting cardiovascular risk in individuals with obesity.

## Materials and methods

This cross-sectional study included 368 patients aged 18 years and older, with BMI of ≥30 kg/m^2^, without known cardiac disease, and was admitted to the Family Medicine outpatient clinic of the S.B.Ü. Kartal Dr. Lütfi Kırdar City Hospital between November 2019-January 2020. The sociodemographic information of the participants, who read and signed the informed consent form, was obtained, and their chronic diseases and drug consumption status were determined. Physical examinations of participants were made, and height, weight, waist circumference, hip circumference, and blood pressure measurements were taken. Remove, blood pressure measurements from both arms after the patient was rested for 10 minutes were obtained, with their averages. The weight and height of participants were measured with bare feet and wearing light clothing. Waist circumference was measured at the thinnest part of the waist, and hip circumference was measured at the widest part of the hips. Fasting plasma glucose, hemoglobin A1c, low-density lipoproteins, high-density lipoprotein (HDL), triglyceride, and total cholesterol values were measured from the blood tests of participants. Abdominal obesity is defined as a waist circumference of ≥94 cm in males and ≥80 cm in females [8]. BMI and ABSI are calculated according to the formula: BMI = weight (kg)/height (m)^2^ and ABSI = waist circumference (cm)/(BMI^2/3^*height^1/2^) [2,4].

Cardiovascular risks of participants were calculated from the pooled cohort equations risk calculator (PRCAE), Framingham, and systematic coronary risk evaluation (SCORE) risk calculation scales. In PRCAE and Framingham risk scale, variables were evaluated such as age, gender, total cholesterol, high-density lipoprotein (HDL), systolic blood pressure, smoking, and antihypertensive treatment and diabetes history, whereas the SCORE risk scale evaluated the variables such as age, gender, total cholesterol, systolic blood pressure, and smoking [9-11]. PRCAE of <5% is considered low risk, 5-7.4% as medium risk, and ≥7.5 as high risk [9]. The calculated coronary event risk is considered to be in Framingham score of <10%, medium risk in 10%-20%, and high risk in >20% [10]. The percentage of determining risk is considered low risk in SCORE of <1%, medium risk 1%-4%, high risk in 5%-9%, and very high risk in ≥10% [11]. Patients with complicated diabetes, severe chronic kidney disease (glomerular filtration rate (GFR) of <30 ml/min/1.73 m^2^), and clinically or imaging-proven cardiovascular diseases are considered at very high risk, and those with uncomplicated diabetes and moderate chronic kidney disease (GFR 30-59 ml/min/1.73m^2^) are considered at high risk without the need for scoring [12].

Study data were analyzed in IBM Corp. Released 2012. IBM SPSS Statistics for Windows, Version 21.0. Armonk, NY: IBM Corp. Descriptive criteria (frequency, percentage, mean, median, standard deviation, and 25^th^-75^th^ percentiles) were used in the analysis. The Kolmogorov-Smirnov was applied as normality test. The Pearson and Spearman correlation tests were used to evaluate the relationships between continuous variables. The Kruskal-Wallis and Mann-Whitney were also used. A p-value of <0.05 was considered significant.

## Results

Of the 368 participants, 302 (82.1%) were females, and 66 (17.9%) were males with a mean age of 46.2 ± 12.0 years. The sociodemographic characteristics of the participants are shown in Table 1. The mean age of the female patients was 46.8 ± 11.9 years, and that of the males was 43.3 ± 12.3 years. The chronic diseases of participants were determined as had diabetes mellitus in 119 (32.3%), hypertension in 121 (32.9%), hyperlipidemia in 277 (75.3%), and obstructive sleep apnea syndrome in five (1.4%) patients. The drugs that are used by the participants include metformin in 125 (34.0%), other oral antidiabetics in 37 (10.1%), insulin 16 (4.3%), antihypertensives in 110 (29.9%), and acetylsalicylic acid in 17 (4.6%) patients. The median measured systolic blood pressure of the participants was 120.0 (110.0-130.0) mmHg, and the median diastolic blood pressure was 70.0 (70.0-88.8) mmHg.

**Table 1:** Sociodemographic characteristics of the participants.

n	368
Age (year)	46.2 ± 12.0
Female (%)	82.1
Male (%)	17.9
Education Status	
Primary school and below (%)	49.2
Secondary school-high school (%)	33.7
University (%)	17.1
Working Status	
Employee and student (%)	30.7
Not working (%)	69.3
Marital Status	
Married (%)	77.2
Single (%)	22.8
Cigarette Consumption	
No/Ex-smoker (%)	78.0
Yes (%)	22.0
Childhood Obesity	
No (%)	66.8
Yes (%)	33.2
Familial Obesity	
No (%)	30.4
Yes (%)	69.6

The anthropometric measurements of the participants revealed a median height of 160.0 (156.0-168.0) cm, a median weight of 97.9 (88.8-112.4) kg, a median waist circumference of 115.0 (106.3-126.8) cm, and a median BMI of 37.6 (34.0-42.5) kg/m^2^. Mild obesity was determined in 125 (34.0%), moderate obesity in 110 (29.9%), morbid obesity in 112 (30.4%), and super obesity in 21 (5.7%) patients. Of the female participants, 102 (33.8%) were mildly obese, 88 (29.1%) were moderately obese, 94 (31.1%) were morbidly obese, and 18 (6.0%) were super obese. Whereas of the male participants, 23 (34.8%) were mildly obese, 22 (33.7%) were moderately obese, 18 (27.3%) were morbidly obese, and three (4.5%) were super obese. The median BMI of female participants was 37.6 (34.0-42.6) kg/m^2^. The mean BMI of male patients was 38.1 ± 6.1 kg/m^2^. No significant difference was found between the mean BMI of the female and male participants (p = 0.441).

The median ABSI of the participants was 0.0816 (0.0775-0.0849). The median of the Framingham risk score of the participants was 6.2 (2.3-14.2), whereas 2.8 (1.2-7.3) in the PRCAE and 1.0 (0.0-2.0) in the SCORE. The distribution of participants according to the risk groups is shown in Table 2.

**Table 2:** Distribution of the participants by risk groups. PRCAE: pooled cohort equations risk calculator, SCORE: systematic coronary risk evaluation

	n (%)
Framingham	
Low risk	234 (63.6)
Medium risk	81 (22.0)
High risk	53 (14.4)
PRCAE	
Low risk	237 (64.4)
Medium risk	41 (11.1)
High risk	90 (24.5)
SCORE	
Low risk	119 (32.3)
Medium risk	114 (31.0)
High risk	127 (34.5)
Very high risk	8 (2.2)

A negative correlation was found between ABSI and BMI (r = −0.107, p = 0.040). A positive correlation was found between ABSI and Framingham risk score and PRCAE risk score (r = 0.297, p = 0.000 and r = 0.305, p = 0.000, respectively). A significant relationship was found between ABSI and Framingham, PRCAE, and SCORE risk groups of the participants (p = 0.000, p = 0.000, and p = 0.000, respectively). The relationship between the cardiovascular risk groups of the participants and ABSI is shown in Table 3.

**Table 3:** Relationship between cardiovascular risk groups of the participants and ABSI. (1a) versus (2a) post hoc p = 0.028; (1a) versus (3a) post hoc p = 0.000; (2a) versus (3a) post hoc p = 0.196. (1b) versus (2b) post hoc p = 0.000; (1b) versus (3b) post hoc p = 0.000; (2b) versus (3b) post hoc p = 1.000. (1c) versus (2c) post hoc p = 0.106; (1c) versus (3c) post hoc p = 0.000; (1c) versus (4c) post hoc p = 0.153; (2c) versus (3c) post hoc p = 0.719; (2c) versus (4c) post hoc p = 0.999; (3c) versus (4c) post hoc p = 1.000. ABSI: a body shape index, PRCAE: pooled cohort equations risk calculator, SCORE: systematic coronary risk evaluation

	ABSI	P-value
Framingham Low risk (1a)	0.0804 (0.0765-0.0840)	0.000
Framingham Medium risk (2a)	0.0836 (0.0787-0.0854)	
Framingham High risk (3a)	0.0831 (0.0807-0.0868)	
PRCAE Low risk (1b)	0.0801 (0.0762-0.0840)	0.000
PRCAE Medium risk (2b)	0.0841 (0.0813-0.0861)	
PRCAE High risk (3b)	0.0830 (0.0805-0.0863)	
SCORE Low risk (1c)	0.0797 (0.0756-0.0836)	0.000
SCORE Medium risk (2c)	0.0815 (0.0786-0.0844)	
SCORE High risk (3c)	0.0828 (0.0790-0.0859)	
SCORE Very high risk (4c)	0.0841 (0.0804-0.0890)	

## Discussion

Of the 368 patients in the study, 302 (82.1%) were females, and 66 (17.9%) were males. The average age of participants was 46.2 ± 12.0 years. The median BMI of participants was 37.5 (34.0-42.4) kg/m^2^. The median ABSI of participants was 0.0816 (0.0775-0.0849). A positive correlation was found between ABSI and Framingham risk score and PRCAE risk score (r = 0.297, p = 0.000 and r = 0.305, p = 0.000, respectively). A significant relationship was found between ABSI and Framingham, PRCAE, and SCORE risk groups (p = 0.000, p = 0.000, and p = 0.000, respectively).

Various risk calculation systems are used to determine the risk of cardiovascular diseases. Framingham, SCORE, and PRCAE risk calculation models are some of these risk calculation systems. The literature review revealed various studies that showed the effectiveness of ABSI in predicting cardiovascular disease risk and mortality. Our study in patients with obesity, of which most were females with high cardiovascular disease risk, revealed a statistically significant relationship between ABSI and the Framingham, SCORE, and PRCAE risk calculation models.

The prevalence of obesity is increasing worldwide, and obesity-related mortality and morbidity are also increasing. The development of cardiovascular diseases is closely associated with increased adiposity [13]. Metabolic diseases and mortality rates are higher in individuals with abdominal obesity. Various studies showed that the use of waist circumference or ABSI instead of BMI in evaluating abdominal obesity might give more accurate results to determine the mortality risk [14,15]. Krakauer et al. defined ABSI using the National Health and Nutrition Examination Survey data from the United States of America [16] and revealed a relationship between ABSI and cardiovascular mortality by following the patients for a long follow-up period [17].

Mameli et al. studied overweight and obesity in children and adolescents aged 2-18 years and revealed that ABSI was an anthropometric measurement that was significantly associated with cardio-metabolic risk factors [1]. Bertoli et al. revealed that ABSI was a predictor of the risk of death in their cohort study, in which they followed for five years the group of patients aged 18 years and over, with BMI of >18.5 kg/m^2^, and 72% were female patients. They revealed a stronger relationship of ABSI with cardiovascular risk factors than BMI. ABSI was associated with all risk factors independent of BMI and is more valuable together with BMI [15]. Lee et al. revealed a higher power of ABSI in predicting mortality risk than BMI and waist circumference [4]. Similarly, Chung et al. revealed that the ABSI z-score was stronger than BMI and waist circumference in predicting cardiovascular mortality [18].

Grant et al. investigated five-year mortality rates in their study and revealed that 4.3% of participants died due to cardiovascular causes and 3.6% due to cancers. They revealed higher mortality rates due to obesity-related cardiovascular diseases and cancers in both genders and a significant positive relationship between ABSI and mortality according to BMI and waist circumference [19].

Gomez-Marcos et al. examined vascular structures to determine cardiovascular risk in their study. In addition to anthropometric measurements, they looked at carotid intima-media thickness and revealed a significant relationship between ABSI and vascular structure and function measurements [20].

The power of ABSI in determining cardiovascular mortality has not yet been fully elucidated. Some conducted studies to determine the role of ABSI in predicting the risk of cardiovascular death examined the relationship between ABSI and cardio-metabolic risk factors and the Framingham risk calculation system [21-23]. Wang et al. revealed that the percentage of participants with moderate and high cardiovascular risk according to the Framingham risk calculation was significantly higher in males, BMI was unsafe in estimating cardiovascular risk, and ABSI was the best predictor for males [21]. Zakri et al. included 120 patients with type 2 diabetes and did not find a significant relationship between the cardiovascular risk groups according to the Framingham risk score and ABSI [22]. Moon et al. revealed that cardiovascular disease rates were higher in patients with a higher ABSI Z-score, and ABSI was more closely associated with cardiovascular disease risk compared to BMI and waist circumference [23].

The Framingham risk-calculation system is frequently used and valuable in demonstrating cardiovascular risk; however, it has some limitations, including its limited use in young patients. Studies showed that the Framingham risk-calculation model shows the risk as low in some populations and high in some populations [24]. The high rate of female patients and the lack of participants with normal BMI are the limitations of our study.

## Conclusions

Our study used SCORE and PRCAE risk calculation systems as well as the Framingham risk calculation system to evaluate cardiovascular risk. The cardiovascular risk calculation models were compared with ABSI and revealed their effectiveness in demonstrating cardiovascular mortality. Our study results revealed a significant association of the score obtained according to ABSI with the Framingham, PRCAE, and SCORE scoring systems in determining cardiovascular risk, which suggests its effectiveness in predicting cardiovascular risk. Larger-scale clinical studies that involve patients with a BMI of <30 kg/m^2^ are needed to confirm the role of ABSI in demonstrating cardiovascular risk.
